# Endovascular treatment of a descending thoracic aorta aneurysm in a patient with right sided aortic arch: a case report

**DOI:** 10.1186/s42155-025-00526-1

**Published:** 2025-05-05

**Authors:** Nefeli Ntinou, Panagiotis Petaloudis, Dimitra Tachmetzidi Papoutsi, Vasileios Panou, Myrto Papadopoulou, Dimitrios Tomais, Ioannis Kalogeropoulos, Theodoros Kratimenos

**Affiliations:** Interventional Radiology Department, Evangelismos General Hospital of Athens, Athens, Greece

**Keywords:** Right-sided aortic arch, Aneurysm, TEVAR, New technologies

## Abstract

**Backround:**

Right-sided aortic arch is a rare congenital variant. The Edwards classification describes three types of right sided-aortic arch: right aortic arch with aberrant left subclavian artery, right aortic arch with mirror image branching, and right aortic arch with isolation of the left subclavian artery.

Aneurysms associated with right sided aortic arch are rare.

Pain is the commonest presenting symptom, but due to the anatomy of the right aortic arch, the symptoms may be atypical, as dysphagia.

We present a case of a challenging endovascular repair in a patient with aneurysm of descending thoracic aorta and right aortic arch.

**Case presentation:**

Α 55 year old patient was admitted in our hospital with chest pain.

After the initial clinical and laboratory workout that was negative for acute coronary syndrome, Computed Tomography Angiography revealed an aneurysm of the descending aorta 10,3 cm in width, and a right sided aortic arch (Edwards’ classification).

Endovascular repair was selected as the treatment option of choice.

Technically the endografting was challenging, firstly because of the right sided aortic arch, secondly because the four aortic branches originate independently. In order to identify the orifices of arch vessels during the angiography, brachial access in both upper extremities was achieved.

In this way, it was possible to correctly deploy the thoracic aortic stent graft.

No endoleaks were observed in the final angiography.

Postoperative Computed Tomography Angiography 10 months after the operation showed no endoleaks.

**Conclusion:**

This case indicates that TEVAR is feasible as a treatment option in patients with right-sided aortic arch, even though technically is challenging.

However more evidence-based data are needed to certify long-term safety and efficacy of endovascular repair in treatment of thoracic aortic aneurysm associated with right-sided aortic arch.

## Background

Right-sided aortic arch is a rare congenital variant with an incidence of 0,1%. A higher proportion of patients are male. This variation is a result of alterations in the normal embryonic development with regression of the left fourth arch or the left dorsal aorta while the right dorsal aorta remains patent. The Edwards classification describes three types of right sided-aortic arch: right aortic arch with aberrant left subclavian artery, right aortic arch with mirror image branching, and right aortic arch with isolation of the left subclavian artery [1–3].

Aneurysms associated with right sided aortic arch are rare. Pain is the commonest presenting symptom and is either felt in the chest, epigastrium or back. Due to the anatomy of the right aortic arch, the symptoms may be atypical, as dysphagia [1–4].

We present a case of a challenging endovascular repair of a descending thoracic aneurysm in a patient with right sided aortic arch (Edwards classification type II: with left aberrant subclavian artery).

### Case presentation

A 55 year old male presented to our hospital with chest pain.

**Fig. 1:**
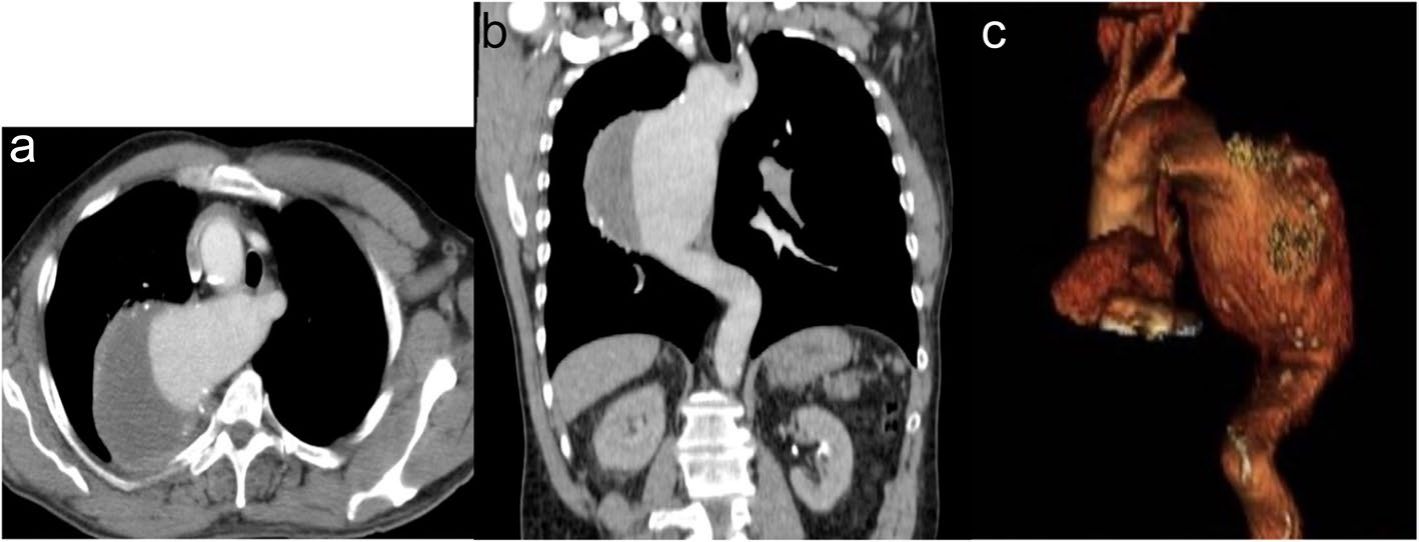
Preoperative CTA: **a**) axial reconstruction of right sided aortic arch and the origin of left common carotid artery and retroesophageal left subclavian artery **b**) coronal reconstruction of the aneurysm of descending aorta c) volume rendering

The patient was hemodynamically stable. Clinical and laboratory workout was negative for acute coronary syndrome, and a Computed Tomography Angiography (CTA) was performed. CTA demonstrated a descending aorta aneurysm and a right-sided aortic arch. The aneurysm measured 10,3 cm in width and 11,8 cm in length. The four aortic branches originated independently and were branching in this order: left common carotid artery (aberrant), right common carotid artery, right subclavian artery (aberrant) and retroesophageal left subclavian artery (Edwards classification type II: with left aberrant subclavian artery). Both vertebral arteries had the expected origin, from left and right subclavian arteries respectively Fig. 1.

A dedicated aortic team consisting of interventional radiologists, cardiac surgeons and anesthesiologists decided that thoracic endovascular repair (TEVAR) was the treatment of choice. For this case two descending thoracic stent grafts (GORE c-TAG) were used, to exclude the aneurysmatic sac. This device has the unique characteristic of an intermediate stage of deployment, while the stent graft is expanded to approximately 50 percent of its diameter, allowing continuous blood flow throughout the procedure, providing additional opportunities to visualize and refine device positioning. In addition this stent graft system also includes a unique angulation control, available at the intermediate stage and then again after full deployment promoting a 360° wall apposition and seal along the aortic wall and the inner curve of the aorta [6, 7, 8]. The proximal and distal neck diameter was 3,3 cm and 2,6 cm respectively. Thus a 40 mm diameter stent graft was used, leading to approximately 20–30% oversizing, according to the company’s instruction for use.

Procedure was performed under local anesthesia and conscious sedation. Patient was placed in supine position. Left common femoral artery was punctured and cannulated, using a 5 Fr angio-sheath. Right common femoral artery was surgically exposed by the cardiovascular surgery team. Approximately 150 ml of nonionic contrast agent were used. During the procedure 5000 units of heparin were administered intravenously. A lumbar drain was not applicable.

**Fig. 2:**
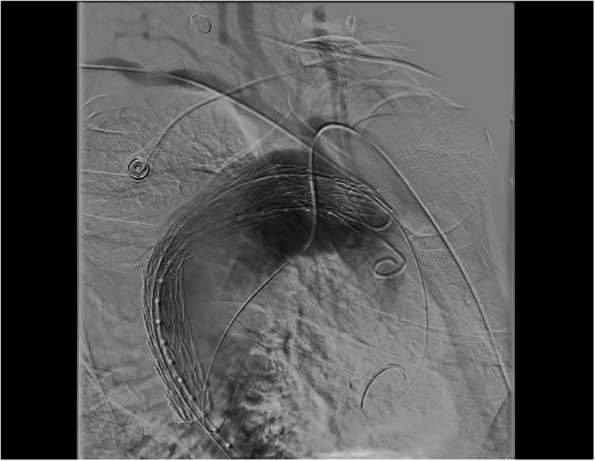
Selective cannulation of both subclavian arteries, during the operation

**Fig. 3:**
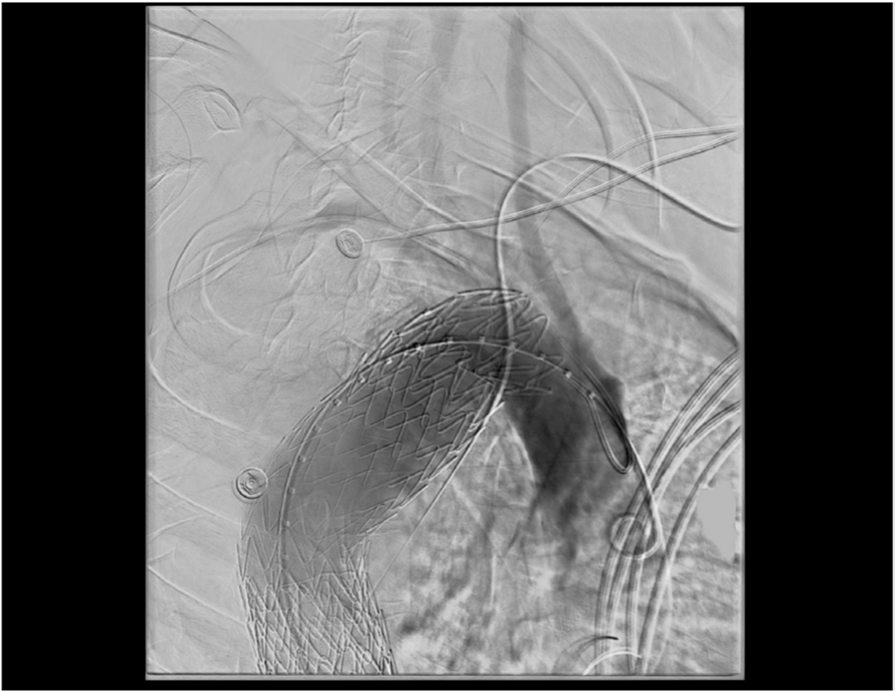
Final post stent-grafting aortogram

Aortography was performed, via femoral route, using a metric pigtail angiographic catheter to extract accurate measurements. However, during the aortography, it was extremely difficult to identify the aortic arch branches despite the use of different angiographic projections, while the patient presented good breath compliance. In order to identify the orifices of the aortic arch vessels, both side brachial arteries had to be punctured, and brachial access was obtained using a 4 Fr angio-sheath on each side. Brachial access was preferred rather than radial because of the better everyday experience of the operating multidisciplinary team. In this way right and left subclavian arteries were selectively cannulated and we were able to better identify the orifices of all 4 aortic branches. Due to short proximal landing zone, coverage of left subclavian orifice with the first stent graft was decided, because thoracic branch endoprosthesis was not commercially available in our country at the time of the operation. After the deployment of the first thoracic stent, a second thoracic stent graft was deployed further in the descending aorta. To ensure full cooptation to the aortic wall a remodeling balloon was inflated in the proximal and distal landing zones. Final post stent-grafting aortogram showed complete exclusion of the aneurysm in the distal aortic arch and descending aorta segment. No endoleaks were observed Figs. 2 and 3.

**Fig. 4:**
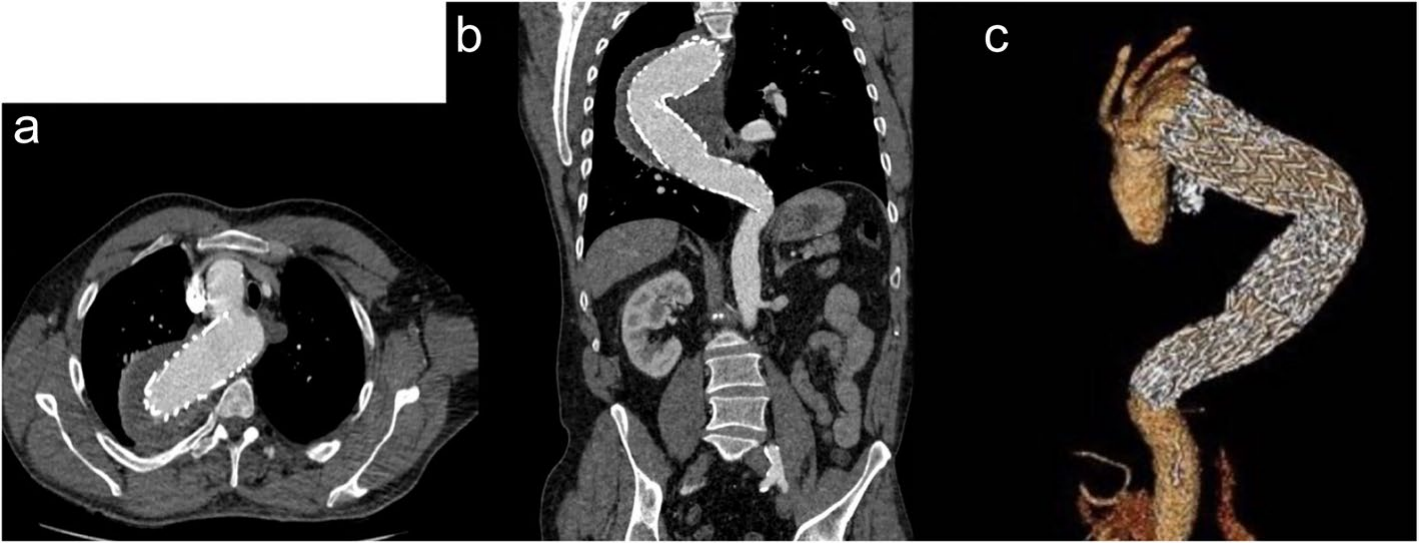
Post- CTA 10 months later: **a**) axial reconstruction of the stent graft in the right sided aortic arch **b**) coronal reconstruction of the stent graft in the descending aorta **c**) volume rendering

Patient had an uneventful post-operative hospitalization and was discharged 4 days later. Follow up CTA 10 months after the procedure showed normally patent aortic stent grafts and complete thrombosis of the aneurysmal sac. There was no evidence of endoleak. The patient remained asymptomatic and never reported any arm claudication Fig. 4.

## Conclusion

Right-sided aortic arch is a rare congenital variant of the vascular thoracic anatomy. Aneurysms in patients with right-sided aortic arch are rare and the published data about endovascular repair are limited [1–3].Thoracic Endovascular Repair for the treatment of descending thoracic aorta aneurysms is a minimally invasive treatment. Preoperative planning and careful delineation of anatomy and possible associated cardiac anomalies are essential to achieve a successful outcome [4, 5, 8].

The endovascular treatment of aneurysms in patients with right-sided aortic arch is challenging, due to the anatomy of the right aortic arch and the possible anatomic variations of the aortic arch branches. Meticulous preoperative planning and identification of possible anatomic variations is crucial for the outcome [2, 3].

In our case the stent-grafting was technically demanding, firstly because of the right sided aortic arch and secondly because the four aortic branches originated independently and in aberrant manner. The selective cannulation of both subclavian arteries, using brachial artery access, was pivotal for the accurate identification of the orifices of all aortic branches. In this way, we were able to correctly deploy the stent-grafts, and successfully exclude the aneurysm sac.

This case indicates that preoperative planning, a dedicated aortic team together with advances and new technologies of the stent-grafts make possible a successful endovascular treatment of such rare pathologies like the right sided descending thoracic aortic aneurysms and could reduce morbidity.

In conclusion the endovascular treatment of aneurysms in patients with right-sided aortic arch is challenging, however our results showed that TEVAR is feasible as a treatment option. None the less, more evidence-based data are needed to certify long-term safety and efficacy of endovascular repair in treatment of thoracic aortic aneurysm associated with right-sided aortic arch [2–4].

## Data Availability

The datasets generated during and/or analysed during the current study are available from the corresponding author on reasonable request.

## Abbreviations

CTA: Computed Tomography Angiography
TEVAR: Thoracic Endovascular Repair
